# *Roseburia intestinalis* Supplementation Could Reverse the Learning and Memory Impairment and m6A Methylation Modification Decrease Caused by 27-Hydroxycholesterol in Mice

**DOI:** 10.3390/nu16091288

**Published:** 2024-04-26

**Authors:** Xuejing Sun, Cui Zhou, Mengwei Ju, Wenjing Feng, Zhiting Guo, Chengyan Qi, Kexin Yang, Rong Xiao

**Affiliations:** Beijing Key Laboratory of Environmental Toxicology, School of Public Health, Capital Medical University, Beijing 100069, China; 1618009@ccmu.edu.cn (X.S.); zhoucui@ccmu.edu.cn (C.Z.); meave_ju@163.com (M.J.); 15810888862@163.com (W.F.); guozhiting51@163.com (Z.G.); shunshun006572@163.com (C.Q.); yangkx5989@163.com (K.Y.)

**Keywords:** 27-hydroxycholesterol, m6A, *Roseburia Intestinalis*, learning and memory ability

## Abstract

The abnormality in N6-methyladenosine (m6A) methylation is involved in the course of Alzheimer’s disease (AD), while the intervention of 27-Hydroxycholesterol (27-OHC) can affect the m6A methylation modification in the brain cortex. Disordered gut microbiota is a key link in 27-OHC leading to cognitive impairment, and further studies have found that the abundance of *Roseburia intestinalis* in the gut is significantly reduced under the intervention of 27-OHC. This study aims to investigate the association of 27-OHC, *Roseburia intestinalis* in the gut, and brain m6A modification in the learning and memory ability injury. In this study, 9-month-old male C57BL/6J mice were treated with antibiotic cocktails for 6 weeks to sweep the intestinal flora, followed by 27-OHC or normal saline subcutaneous injection, and then *Roseburia intestinalis* or normal saline gavage were applied to the mouse. The 27-OHC level in the brain, the gut barrier function, the m6A modification in the brain, and the memory ability were measured. From the results, we observed that 27-OHC impairs the gut barrier function, causing a disturbance in the expression of m6A methylation-related enzymes and reducing the m6A methylation modification level in the brain cortex, and finally leads to learning and memory impairment. However, *Roseburia intestinalis* supplementation could reverse the negative effects mentioned above. This study suggests that 27-OHC-induced learning and memory impairment might be linked to brain m6A methylation modification disturbance, while *Roseburia intestinalis*, as a probiotic with great potential, could reverse the damage caused by 27-OHC. This research could help reveal the mechanism of 27-OHC-induced neural damage and provide important scientific evidence for the future use of *Roseburia intestinalis* in neuroprotection.

## 1. Introduction

As a degenerative brain disorder, Alzheimer’s disease (AD) is caused by neuronal damage in the brain. When aberrant biological signs such as higher levels of tau and amyloid (Aβ) protein in the cerebrospinal fluid and impaired glucose metabolism are observed, pathological alterations in the brain are frequently seen 20 years before symptoms appear. As the disease progresses, the brain is unable to compensate for AD-induced neuronal damage and death, resulting in abnormally high rates of cognitive loss which defies the expectations of normal aging [1]. Although the first drug targeting amyloid-β (Aβ) deposition in the brain of AD patients to alleviate cognitive decline has completed phase III clinical trials successfully [2], the absolute difference in the outcome of this drug compared to placebo intervention is less than the conventionally defined minimum clinically important difference, and the uncleared clinical benefit and the adverse effects such as cerebral edema cannot be ignored. Moreover, its high price and long-term regimen will impose a heavy financial burden [3]. Therefore, Further exploration of the underlying causes of AD is needed to develop drugs that target other causes of AD. Numerous findings suggest that disorders of cholesterol/oxysterol metabolism [4,5,6], intestinal flora [7,8,9], and epigenetic modifications [10,11,12] play important roles in the development of AD; however, it remains to be clarified what roles do they play in the disease’s progression and how they are related to each other.

High peripheral cholesterol levels from both dietary nutrient sources and hepatic metabolism can cause an imbalance in cerebral oxysterol homeostasis, metabolizing an excess level of 27-Hydroxycholesterol (27-OHC) through the blood-brain barrier [13]. Abnormally elevated 27-OHC levels have been recognized as a significant risk factor for both AD and the mild cognitive impairment (MCI) stage of pre-AD. Case-control studies and animal experiments have verified the causal relationship between 27-OHC and MCI [14,15,16]. In the subsequent exploration of how 27-OHC leads to the impairment of learning and memory ability, it was found that the intervention of 27-OHC caused abnormal changes in the intestinal microbiota [17] and epigenetic modification (unpublished data). These findings suggest that the role of intestinal microbiota and epigenetic modification in learning and memory impairment caused by 27-OHC should be further explored.

The gut microbiota is crucial to the development of AD. Abnormal alterations in the gut flora, its metabolites, and related hormones can damage the intestinal and blood-brain barriers, causing amyloidosis deposition in the gut [18,19,20]. At the same time, gut-derived Aβ may be a major source of Aβ in the brain [21]. Recent research has found that anomalous changes in intestinal flora play an important role in the process of 27-OHC leads to cognitive impairment [22], as the results show, 27-OHC intervention could lead to learning and memory impairment in mice with normal gut microbiota, but after the treatment of antibiotic cocktail (ABX) for 4 weeks to deplete the gut microbiota, then give the mice a daily subcutaneous injection of 27-OHC or saline for 3 weeks while remaining the ABX treatment, the 27-OHC treatment couldn’t decrease learning and memory ability compared with the saline-treat group, which made it clear that the gut microbiota mediated the process of 27-OHC impairs learning and memory ability. Following the 27-OHC intervention, fecal 16S rDNA sequencing results in APP/PS1 mice showed a substantial reduction in the relative abundance of *Roseburia* spp. in the gut [17]. As promising probiotics, significant down-regulation of the abundance of *Roseburia intestinalis* and *Roseburia hominins* were observed in both AD populations and AD mouse models compared to healthy controls [23,24], and a mitigating effect on neuroinflammation was observed in colonization studies of *Roseburia* spp. in both mice and rats [25,26]. *Roseburia inulinivorans* is more recognized as a potential psychotropic probiotic [27], which aroused our interest in exploring whether the supplementation of intestinal *Roseburia* spp. could alleviate learning and memory impairment caused by 27-OHC.

Epitranscriptome modifications participated in various biological processes of the AD progression from early dysfunction to dementia, including synaptic deficits and Aβ deposition. N6-methyladenosine (m6A), as the most common RNA modification in mammals and the most varied post-transcriptional alteration of eukaryotic RNA, is highly enriched in the brain and has powerful features of controlling all aspects of mRNA metabolism, from transcription to decay, including splicing, stability, localization, and translation [28], playing an important role in the development of AD [29,30]. Based on the findings that m6A methylated methyltransferases, demethylases, and m6A resultant proteins are located in neuronal dendrites and adjacent to the postsynaptic membrane [30,31], the key role of m6A in synaptic activity attaches a lot of attention, and the relationship between m6A and some of the synapse-related molecules such as PSD-95 [32] and BDNF [33] had been proved in existing researches [34,35]. The current research on the relationship between m6A and AD is still shallow [36], exploring the role of m6A in the progression of AD is undoubtedly an emerging and promising research hotspot, which will provide new options for the treatment of AD. In the preliminary exploration of our group (unpublished data), it was found that the m6A methylation level in the brains of 27-OHC-treated C57BL/6J mice showed a downward trend, while the expression level of the m6A-related modifier METTL4 gene was significantly down-regulated, which suggested that there is a certain relationship between m6A methylation modification and 27-OHC, the disordered 27-OHC level in the body may lead to abnormal changes in m6A methylation level by regulating the m6A methylation modifying enzyme level.

The important role of 27-OHC in the occurrence and development of AD is clear, and it has been hypothesized that m6A methylation modification and *Roseburia intestinalis* may be involved during AD induced by 27-OHC dysregulation. In this study, the aforementioned queries, including whether 27-OHC, *Roseburia intestinalis*, and m6A are related to each other and what role they might play, had to be promptly investigated.

## 2. Materials and Methods

### 2.1. Roseburia Intestinalis Culture

*Roseburia intestinalis* (Deutsche Sammlung von Mikroorganismen und Zellkulturen GmbH, DSMZ-14610, Braunschweig, Germany) was cultured at 37 °C in Modified Reinforced Clostridial Broth Medium (TUOPU BIOL-ENGINEERING, Weihai, Shandong, China) in an anaerobic chamber (Sheldon, Cornelius, OR, USA). The colony forming units (CFU) of *Roseburia intestinalis* were estimated by McFarland standard.

### 2.2. Animal and Experimental Design

**Figure 1:**
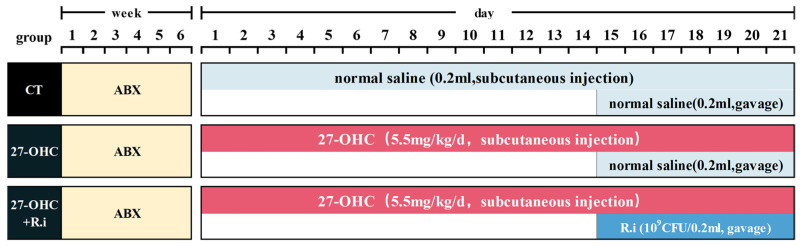
A schematic diagram of protocol design and drug treatment.

The 9-month-old male C57BL/6J mice were purchased from the Laboratory Animal Department of Capital Medical University. All mice were kept under specific-pathogen-free (SPF) conditions with natural lighting (12 h light/dark cycle), humidity (50–55%), and temperature (20–23%). with. standard diet and water ad libitum. All animals were allowed free access to food and water and weighed once a week. All of the mice in this study were given a 2-week conditional diet, followed by 6 weeks of antibiotic cocktail (1 g/L Ampicillin, 1 g/L Neomycin, 1 g/L Metronidazole, and 0.5 g/L Vancomycin, ABX) drinking water treatment to clean the mice’s intestinal flora [37], the antibiotics were renewed every two days. Following ABX treatment, drinking water was switched to regular drinking water, and the mice were randomly divided into three groups (*n* = 6/group), namely the control group, the 27-OHC group, and the 27-OHC + *Roseburia intestinalis* (27-OHC + R.i) group, according to their body weight. The dose of 27-OHC (5.5 mg/kg/d) [17,22] and *Roseburia Intestinalis* (10^9^ CFU/d per mouse) [22,38] was determined as previously described. The control groups underwent subcutaneous saline injection and gavage. The 27-OHC-treated group received constant 27-OHC treatment for 21 days, and the *Roseburia intestinalis* treatment group underwent 7 consecutive days of *Roseburia intestinalis* gavage from the 15th to the 21st day of the 27-OHC injection. Behavioral experiments were conducted after all treatment, then organs were separated and the biological samples were stored in the −80 °C refrigerator for later use. All animal experiment protocols were approved by the Animal Care and Use Committee of Capital Medical University (Beijing, China. NO. AEEI-2022-007), see Figure 1 for details.

### 2.3. Neurobehavioral Tests

#### 2.3.1. Novel Object Recognition Test

To assess short-term memory novel object recognition test was conducted as described previously [39]. In brief, the mice were subjected to an 8-min adaptation experiment in an empty experimental box, and after 24 h of adaptation experiment, two identical objects were placed in two corners of the experimental box and put with the mice for 8 min for training experiments. A total of 24 h after the training experiment, an object in the experiment box was replaced with an object which has a completely different shape, size, and color, and the test experiment was carried out for 8 min. A 75% ethanol solution was used to mask the odor cues after each operation. The recorded frequency and time of exploring novel/old objects can be used to calculate the indexes (novel object recognition index (NORI), frequency identification index (FDI), and time discrimination index (TDI)).

#### 2.3.2. Y Maze Test

The Y-maze spontaneous alternation experiment utilizes the tendency of mice to alternate between alternatives to assess short-term memory capacity in mice [40]. Each arm of the Y maze apparatus is 4 cm wide, and 36 cm long, and the walls are 15.5 cm high. The mice were placed at the end of the same side arm, and the trajectories of the mice in the three arms of the Y maze were recorded for 8 min. After which the mice that had completed the experiment were returned to the cage, and the feces and urine in the Y maze were cleaned up, and sprayed with alcohol to eliminate the residual odor of the mice. Spontaneous alternation (%) = Correct number of times to enter the arm/(number of times to enter the arm − 2) × 100%

#### 2.3.3. Morris Water Maze (MWM) Test

The Morris water maze test (JX business, Shanghai, China) was used to detect the spatial learning ability and memory of mice [41]. The diameter of the maze equipment is 120 cm, the height is 50 cm, the water temperature is 21 ± 1 °C, and an appropriate amount of titanium dioxide is added to the pool to improve the tracking effect of the system on mice. The pool was divided into four quadrants and an escape platform was submerged in one of the quadrants. In the five-day orientation navigation tests, mice were dropped into three quadrants outside the platform and allowed to swim for 90 s to find the platform, and the time when the mouse found the platform was recorded as the escape latency, if no platform was found, the mouse would be guided to the platform and allowed to stay for 15 s, and the recorded escape latency was 90 s. In the space exploration experiment on the sixth day, the platform was removed, the mice were put into the pool from the opposite quadrant of the platform, and they swam freely for the 90 s, and the data such as the number of times the mice crossed the platform were recorded.

### 2.4. Hematoxylin-Eosin (HE) Staining

HE staining was utilized to observe the pathological changes in the brain and colon of mice in each group. In brief, fresh whole brains and colons were harvested and fixed in 4%paraformaldehyde for at least 24 h. All tissues are then transferred to gradient ethanol, dehydrated, and embedded in paraffin. Cut tissue sections (4 μm thick) using a rotary microtome (Leica, Weztlar, Germany). After deparaffinization and rehydration, stain tissue sections with hematoxylin solution (ZKWB-BIO, Beijing, China) for 5 min, then soak in 1% acidic ethanol 5 times, and then rinse with distilled water. It was then stained with eosin solution (ZKWB-BIO, Beijing, China) for 3 min and dehydrated and clarified using alcohol-xylene. The slides are then scanned and analyzed using an automatic section scanner (3DHISTECH Ltd., Gaomi, China).

### 2.5. High-Performance Liquid Chromatography-Mass Spectrometry (HPLC–MS)

The levels of 27-OHC and 24S-hydroxycholesterol (24S-OHC) in the brain tissues were detected by HPLC-MS according to previous studies [16,42]. In brief, 50 μL brain homogenates were prepared for each sample, adding internal standard (50 μL d5-27-OHC/d7-24-OHC mixture) into each tube, subsequently derivatizing the sample with niacin, 4-Dimethylaminopyridine, N,N-Diisopropylcarbodiimide, and Dichloromethane mixtures and then evaporating the contents in a vacuum dryer. The samples were redissolved in 100 μL methanol, after which the supernatants were collected into the loading vials and analyzed by HPLC -MS.

**Figure 2:**
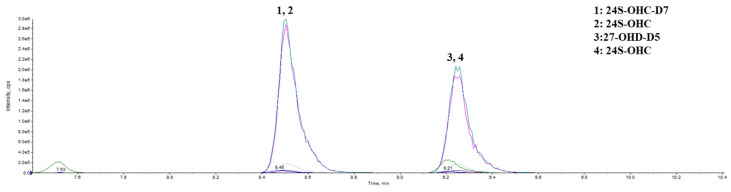
The characteristic chromatogram of the HPLC-MS analysis.

HPLC-MS analysis was conducted on the QTRAP™ 6500 LC-MS/MS system (AB Sciex, Washington, DC, USA). EclipsePlusC18 column (2.1 × 100 mm, 1.8 μm; Agilent, Santa Clara, CA, USA) was used to perform the chromatography. For the LC, mobile phase A (0.1% formic acid in water) and mobile phase B [methanol] were operated with a gradient elution as follows: 0–2.5 min 10% B, 2.5–10.00 min 95% B, 10.00–10.51 min 100% B, and 10.51–13.00 min 10% B at a flow rate of 0.3 ml/min. (Figure 2, Table 1).

### 2.6. Total RNA m6A Modification Level

The m6A level of total RNA in the brain cortex was detected by the EpiQuik m6A RNA Methylation Quantification kit (Epigentek, New York, NY, USA). First, total RNA was isolated from the brain by E.Z.N.A. miRNA Kit R6842 (Omegabio-tek, Guangzhou, China) using the protocol of isolating total RNA according to the manufacturer’s instruction, and then 200 ng of total RNA was taken to detect the m6A level according to the manufacturer’s instruction.

### 2.7. Quantitative Real-Time PCR (RT-PCR)

RT-qPCR was performed to detect the mRNA expression levels of m6A methylation-relate enzymes and synaptic function molecules in the cerebral cortex of mice, where m6A modification-related enzymes include METTL3, METTL4, METTL14, FTO, WTAP, YTDHF-1. Synaptic function molecules include PSD-95 and BDNF.

In brief, the primers were designed specifically by retrieving from the NCBI (Table 2). 1 μg of total RNA of each sample was taken, using the First Strand cDNA Synthesis Kit (ThermoFisher Scientific, Waltham, MA, USA) for reverse transcription. RT-qPCRs were conducted using a KAPA SYBR PCR Kit (Kapa Biosystems, Wilmington, MA, USA) with β-Actin as an internal reference for normalization.

### 2.8. Western Blot

Western blot was performed to detect the protein expression levels of m6A modification-related enzymes, synaptic function molecules in the cerebral cortex, and intestinal barrier function-related proteins in the colon. Thereinto, m6A modification-related enzymes include METTL4, and METTL3, synaptic function molecules include PSD-95, and intestinal barrier function-related proteins include occluding-1 and claudin-1.

In brief, approximately 40 mg of brain cortex or colon tissue was lysed in RIPA buffer (add PMSF). Then homogenizing the tissues and centrifuging (12,000 rpm, 5min, 4 °C) to collect the supernatant and using the bicinchoninic acid (BCA) method to measure the protein concentration. 40 μg protein samples were separated by SDS-PAGE and transferred to PVDF membranes. The antibodies used were as below: β-actin (ABclonal, AC026, 1:50,000, Wuhan, China), METTL4 (bioss, bs-18851R, 1:1000, Beijing, China), PSD-95 (Abcam, ab18258, 1:1000, Cambridge, UK), occludin (Abcam, ab216327, 1:1000, Cambridge, UK), claudin-1 (Abcam, ab180158, 1:1000, Cambridge, UK). Image System Fusion FX (Vilber Lourmat, Marne-la-vallée, France) were used to detect the protein density.

### 2.9. Statistical Analysis

Statistical analysis was processed using SPSS 23.0 (SPSS, Chicago, IL, USA) and GraphPad Prism 7.0.0 software. Data are presented as mean ± SEM. One-way ANOVA with Bonferroni correction for multiple group comparisons (for parametric data) and the Kruskal-Wallis test (for nonparametric data) were used. The body weight and escape latency in the Morris water maze test were analyzed using repeated-measures ANOVA. A level of *p* < 0.05 was considered significant.

## 3. Results

### 3.1. Body Weight and Organ Coefficient Were Not Affected by the 27-OHC Intervention and Roseburia Intestinalis Gavage

To evaluate the specific toxic effect of the 27-OHC intervention and *Roseburia intestinalis* gavage after antibiotic treatment, the body weight of all animals was measured once a week during the intervention period, and the organ coefficients (organ weight/body weight, %) were calculated after recording the absolute organ weights during the deconstruction phase.

**Figure 3:**
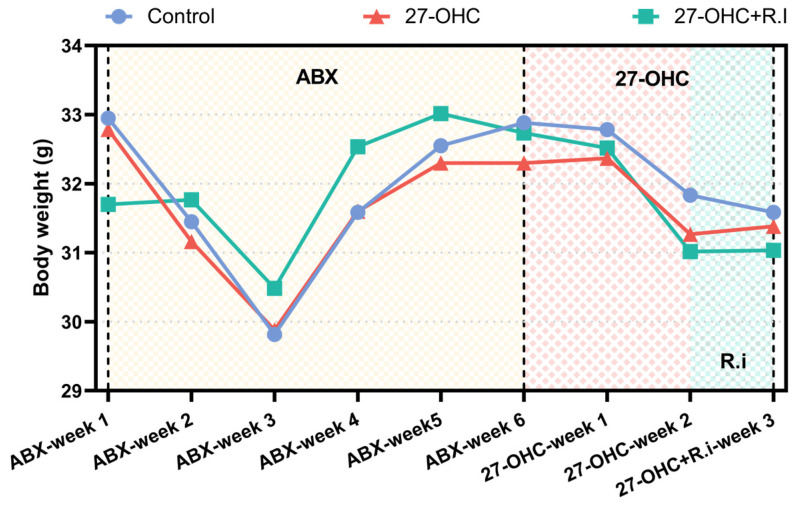
Body weight of C57BL/6J mice in each group. n = 5–6 mice/group. All the data are presented as means.

As the weight change data shows, the mouse weight decreased during ABX treatment, reaching its lowest point at the 3rd week, and then the body weight increased gradually, no statistically significant differences were observed among the three groups during this stage. After ABX treatment, saline, 27-OHC, and *Roseburia intestinalis* were administered. Body weights of all the groups showed a decreased tendency, and the intervention did not result in a significant difference in body weight among the groups (*p* > 0.05) (Figure 3).

**Figure 4:**
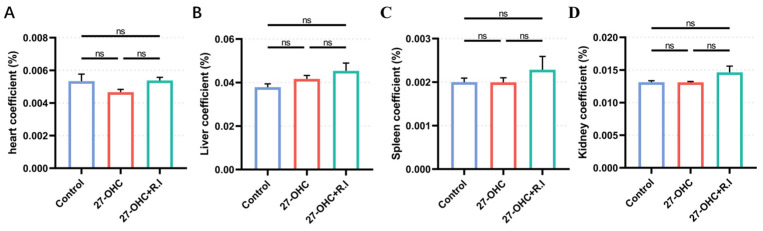
Organ coefficient of C57BL/6J mice in each group. (**A**) heart, (**B**) liver, (**C**) spleen, (**D**) kidney. *n* = 5–6 mice/group, All the data are presented as mean ± SEM. ns *p* > 0.05.

There were also no significant differences observed in the coefficients of the heart, liver, spleen, and kidney among the three treated groups (*p* > 0.05) (Figure 4).

### 3.2. Roseburia Intestinalis Intervention Reversed the Impaired Learning and Memory Ability Caused by 27-OHC Treatment

The novel object recognition, Y maze, and Morris water maze tests were carried out to evaluate the effects of 27-OHC and *Roseburia intestinalis* treatment on the learning and memory ability of mice.

**Figure 5:**
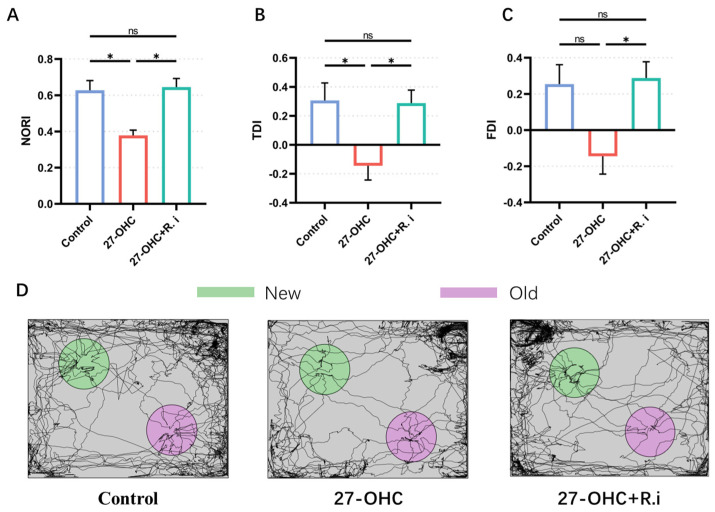
Results of Novel Object Recognition test. (**A**) Novel object recognition index (NORI), (**B**) Time discrimination index (TDI), and (**C**) Frequency identification index (FDI) of C57BL/6J mice in each group. (**D**) The path of mice in the process of the training test. *n* = 3 mice/group. All the data are presented as means ± SEM. * *p* < 0.05. ns *p* > 0.05.

In the novel object recognition test, the results showed that there were significant statistical differences in NORI (F = 8.082, *p* = 0.0098), FDI (F = 4.948, *p* = 0.0355), and TDI (F = 5.004, *p* = 0.0346) among the three groups. Compared with the control group, the NORI (*p* = 0.025) and TDI (*p* = 0.0475) in the 27-OHC group were significantly reduced, and the FDI (*p* = 0.0839) showed a downward trend, the *Roseburia intestinalis* treatment reversed the decrease in NORI (*p* = 0.0132), FDI (*p* = 0.0476), and TDI (*p* = 0.0471) caused by 27-OHC, and there was no significant difference between the control group and the 27-OHC+*R.i* group (*p* > 0.99) (Figure 5).

**Figure 6:**
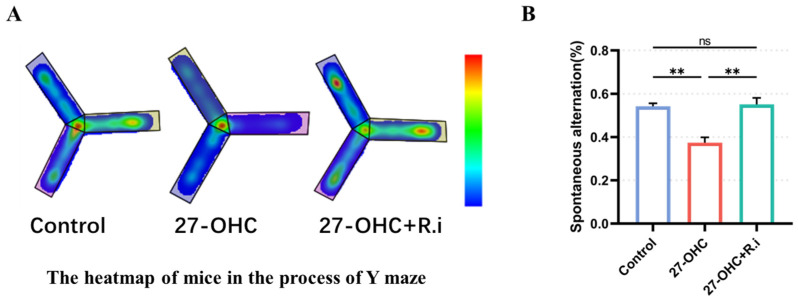
Results of the Y maze test. (**A**) The heatmap of mice in the process of exploring the Y maze. (**B**) Time spontaneous alternation of C57BL/6J mice in each group. n = 6 mice/group. *n* = 4–5 mice/group. All the data are presented as means ± SEM. ** *p* < 0.01. ns *p* > 0.05.

In the Y maze test, the results showed that there were significant statistical differences in the spontaneous alternation rate of mice (F = 18.28, *p* = 0.0005), among which the spontaneous alternation rate of mice in the 27-OHC group was significantly lower than that in the control group (*p* = 0.0016), while *Roseburia intestinalis* treatment could significantly improve the spontaneous alternation rate of mice caused by 27-OHC (*p* = 0.0011), and there was no significant difference when compared with the control group (*p* > 0.9999) (Figure 6).

**Figure 7:**
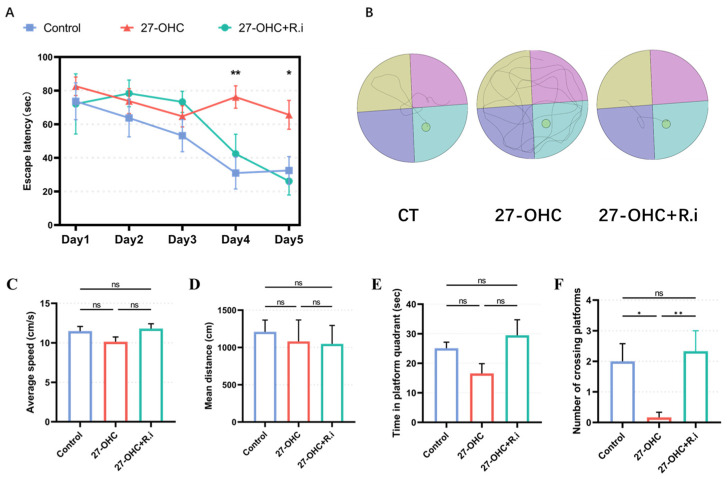
Results of Morris Water Maze test. (**A**)The escape latency of mice changes with time during the training phase. (**B**) The path of mice in the process of exploring the platform. (**C**) Average speed. (**D**) Mean distance. (**E**) Time in platform quadrant. (**F**) Number of crossing platforms. *n* = 3–6 mice/group. All the data are presented as means ± SEM. * *p* < 0.05. ** *p* < 0.01. ns *p* > 0.05.

As the outcome of MWM test showed, during the orientation navigation tests, Time factors (F = 14.68, *p* < 0.001) and inter-group treatment factors (F = 6.123, *p* = 0.0027) had remarkable effects on the escape latency of mice, and there was an interaction between time factors and treatment factors between groups (F = 4.064, *p* = 0.0002). Further analysis showed that the escape latency of the Control group (*p* = 0.0064) and the 27-OHC+*R.i* group (*p* = 0.0027) was significantly reduced on the fourth day compared with that on the first day, while the escape latency of 27-OHC did not change in the positioning navigation experiment (*p* > 0.1). Prominent differences existed among the three groups on the fourth day and fifth day, in which the control group and 27-OHC+*R.i* group had a lower escape latency than that of the 27-OHC group (Figure 7A,B).

The results of space exploration experiment showed that there was a noteworthy statistical difference in the number of crossings of the platform in the Morris water maze experiment of mice in each group (F = 9.955, *p* = 0.0052) (Figure 7F), among which the number of crossings of the platform was lower in the 27-OHC group than in the control group (*p* = 0.0221), while the number of crossings of the platform in the 27-OHC treatment was improved after *Roseburia intestinalis* treatment (*p* = 0.0089). The average speed (F = 2.283, *p* = 0.1047) (Figure 7C), mean distance (F = 0.1156, *p* = 0.8616) (Figure 7D), and time in platform quadrant (F = 2.932, *p* = 0.0864) (Figure 7E) had no significant differences among the three groups in the Morris water maze experiment (Figure 7).

### 3.3. Roseburia Intestinalis Intervention Improved the 27-OHC-Induced Morphology Change in the Brain

**Figure 8:**
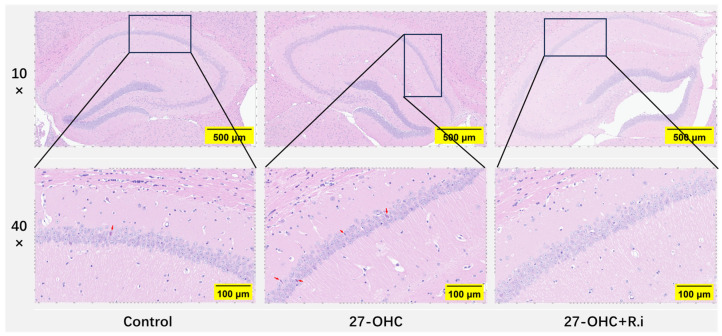
HE staining for brain tissue in C57BL/6J in each group. 10× (scale bar = 500 μm), 40× (scale bar = 100 μm), red arrows: nuclei pyknosis.

HE staining was employed to explore the effect of treatment on the histomorphology of the brain. As presented in Figure 7, the arrangement of neurons in the hippocampal CA1 region was loose and disordered in the 27-OHC group compared with the Control group, and an increase of nuclei pyknosis was observed in the hippocampal CA1 region. In comparison, the brain histomorphology of 27-OHC + *R.i* group exhibited more regular neuron arrangement and fewer nuclei pyknosis in the hippocampal CA1 region compared to the 27-OHC group (Figure 8).

### 3.4. Roseburia Intestinalis Intervention Could Restore the Decrease of Synapse-Related Molecules Caused by 27-OHC in Brain Cortex

**Figure 9:**
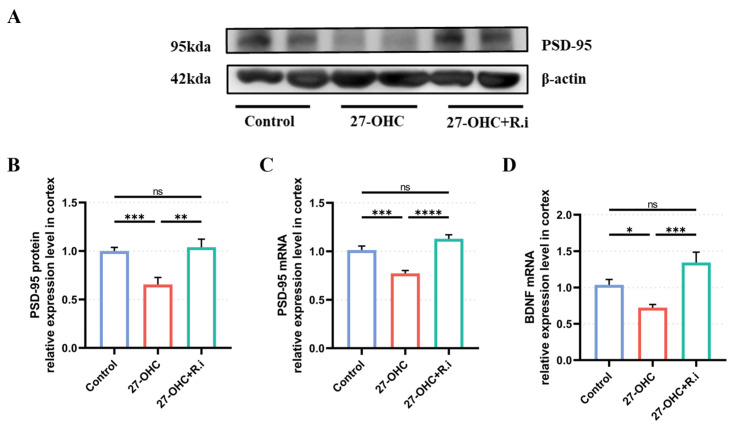
Expression level of synapse-related molecules in C57BL/6J mice brain cortex. (**A**) Western blot results of PSD-95. (**B**) PSD-95 protein expression level. (**C**) PSD-95 mRNA expression level. (**D**) BDNF mRNA expression level. *n* = 4–5 mice/group. All the data are presented as means ± SEM. * *p* < 0.05. ** *p* < 0.01. *** *p* < 0.001. **** *p* < 0.0001. ns *p* > 0.05.

For evaluating the effects of 27-OHC intervention and *Roseburia intestinalis* gavage on synaptic function-related molecules after antibiotic treatment, RT-qPCR, and Western blot were used to detect the levels of synapse-related molecular genes and proteins such as PSD-95 and BDNF in the cerebral cortex of mice. The results showed that there were statistically significant differences in the expression levels of the PSD-95 gene (F = 22.79, *p* < 0.0001) and protein (F = 11.8, *p* = 0.0003) in the cerebral cortex of mice in different groups, in which the expression levels of PSD-95 gene (*p* = 0.0003) and protein (*p* = 0.0007) in the27-OHC group were lower than those in the control group, while the reduced expression level of PSD-95 gene (*p* = 0.0007) and protein (*p* = 0.0012) in the brain cortex caused by 27-OHC treatment could be reversed by *Roseburia intestinalis* treatment (Figure 9A–C). Besides, the expression level of the BDNF gene in the cerebral cortex among each group was also prominently different. The 27-OHC group had a lower level of BDNF gene expression level than that of the control group (*p* = 0.0138), while the expression level of BDNF was in the 27-OHC + *R.i* group was higher than that of the 27-OHC group (*p* = 0.0004) (Figure 9D).

### 3.5. Roseburia Intestinalis Complementation Reversed the Abnormal Change in m6A Methylation Modification Level and Its Related Enzymes Caused by 27-OHC

**Figure 10:**
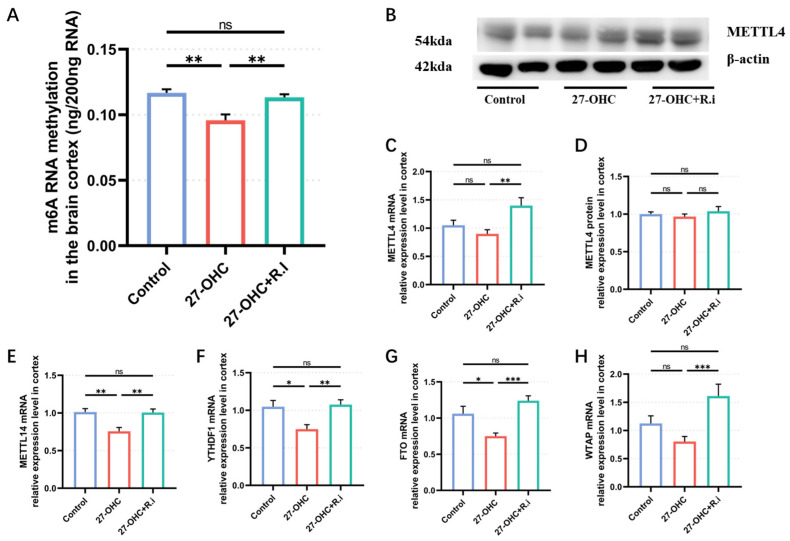
Levels of the m6A modification and its related enzyme expression. (**A**) m6A methylation in the brain cortex (ng/200ng RNA). (**B**) Western blot result of METTL4. (**C**) METTL4 mRNA relative expression level in brain cortex. (**D**) METTL4 protein expression level in the brain cortex. (**E**) METTL14 mRNA relative expression level in the brain cortex, (**F**) YTHDF-1 mRNA relative expression level in the brain cortex. (**G**) FTO mRNA relative expression level in the brain cortex. (**H**) WTAP mRNA relative expression level in the brain cortex. *n* = 4–5 mice/group. All the data are presented as means ± SEM. * *p* < 0.05. ** *p* < 0.01. *** *p* < 0.001. ns *p* > 0.05.

The total RNA m6A methylation modification level in the brain cortex was determined by the EpiQuik m6A RNA Methylation Quantification kit. As the result shows, 27-OHC and *Roseburia intestinalis* intervention caused a prominently different total RNA m6A modification level in the brain cortex of each group (F = 12.14, *p* = 0.0028). Among them, the 27-OHC group had a lower m6A modification level in the brain cortex than the control group (*p* = 0.0033), and the m6A modification level in the brain cortex of the 27-OHC+*R.i* group was significantly higher than that in the 27-OHC group (*p* = 0.01) (Figure 10A). RT-qPCR and Western blot were used to detect the levels of m6A modification-related enzymes genes and proteins in the cerebral cortex of mice, such as METTL4, METTL14, FTO, etc. The results show that the genes expression level of METTL14 (F = 9.863, *p* = 0.0003), YTHDF1 (F = 6.838, *p* = 0.0026), and FTO (F = 8.949, *p* = 0.0006) were remarkably different among groups, and all of them shown a similar trend, the genes expression level of METTL14, YTHDF1, and FTO in the 27-OHC group (*p* = 0.0012, *p* = 0.0135, *p* = 0.0394) were significantly lower than the control group and higher than the 27-OHC+*R.i* group (*p* = 0.0013, *p* = 0.0041, *p* = 0.0004) (Figure 10E–G). The gene expression level of METTL4 in the brain cortex of 27-OHC+*R.i* group was significantly higher than the 27-OHC group (*p* = 0.0018), however, the protein expression level of METTL4 in the brain cortex showed no remarkable change among the three groups (K = 0.2339, *p* = 0.8896) (Figure 10B–D). The WTAP gene’s mRNA expression level in the 27-OHC + *R.i* group was significantly higher than that in the 27-OHC (*p* = 0.0043), but there was no difference revealed between the control group and the 27-OHC group (*p* = 0.7436).

### 3.6. Roseburia Intestinalis Complementation Improved the Level of 24S-OHC in the Brain

**Figure 11:**
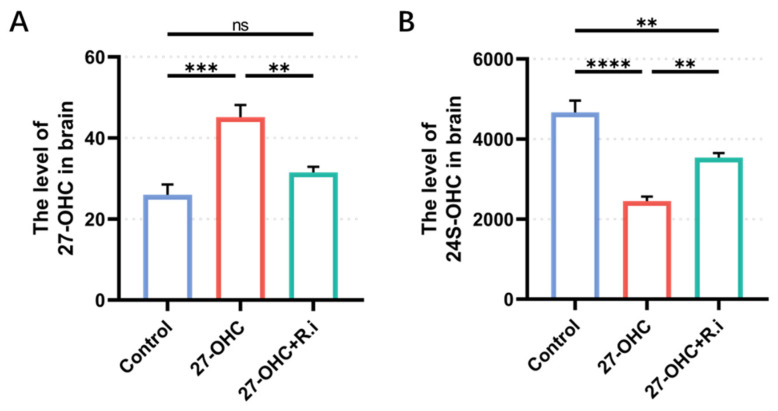
The level of 27-OHC and 24S-OHC in C57BL/6J mice brain. (**A**) The level of 27-OHC in the brain. (**B**) The level of 24S-OHC in the brain. *n* = 4 mice/group. All the data are presented as means ± SEM. ** *p* < 0.01. *** *p* *<* 0.001. **** *p* < 0.0001. ns *p* > 0.05.

24S-OHC is regarded as a protective factor for AD [43], the vivo studies results shows that the 24S-OHC treatment in mice has neuroprotective effect [16]. The levels of 27-OHC and 24S-OHC in mouse brain tissue were determined by HPLC-MS, and the results showed remarkable differences in the levels of 27-OHC (F = 17.09, *p* = 0.0009) and 24S-OHC (F = 32.87, *p* < 0.0001) in the brain tissues of mice in each group. Compared with the control group, the 27-OHC group (*p* = 0.0009) had higher levels of 27-OHC, while 27-OHC + *R.i* group had a significantly lower level of 27-OHC compared with the 27-OHC group (*p* = 0.0089) and had no significant difference compared with the control group (*p* = 0.4006) (Figure 11A). The level of 24S-OHC in brain tissue decreased prominently after 27-OHC treatment (*p* < 0.0001) compared with the control group, and the 24S-OHC level decreased by *Roseburia intestinalis* treatment to a certain extent (*p* = 0.0080), but there was still a notable difference between the control group and the 27-OHC + *R.i* group (*p* = 0.0066) (Figure 11B).

### 3.7. Roseburia Intestinalis Intervention Saved the Impaired Colon Barrier Function Caused by 27-OHC

**Figure 12:**
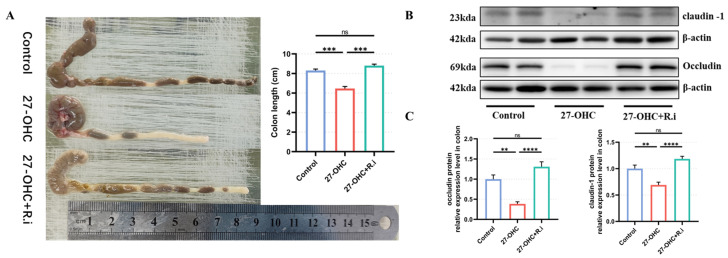
The colon barrier function of C57BL/6J mice. (**A**) The colon length, (**B**) Western blot results of occludin and claudin-1. (**C**) The protein expression level of occludin and claudin-1 in the colon tissues of mice. *n* = 4–5 mice/group. All the data are presented as means ± SEM. ** *p* < 0.01. *** *p* < 0.001. **** *p* < 0.0001, ns *p* > 0.05.

The length of freshly picked mouse colons in each group was measured by a steel ruler. The results showed that 27-OHC intervention and *Roseburia intestinalis* gavage after antibiotic treatment significantly affected the colon length of mice in each group (F = 17.70, *p* = 0.0005). 27-OHC treatment resulted in a remarkable reduction in colon length compared with the control group (*p* = 0.0031), while gavage with *Roseburia intestinalis* reversed the negative effect of 27-OHC on colon length (*p* = 0.0005) (Figure 12A).

The expression levels of occludin, claudin-1, and other intestinal barrier-associated proteins in the mouse colon were detected using Western blot. The results showed that antibiotic treatment followed by 27-OHC intervention and the *Roseburia intestinalis* gavage would result in prominent differences in the protein expression levels of occludin (F = 24.33, *p* < 0.0001) and claudin-1 (F = 21.4, *p* < 0.0001). The 27-OHC group showed lower protein expression levels of occludin (*p* = 0.0025) and claudin-1 (*p* = 0.0017) compared to the control group, whereas the *Roseburia intestinalis* intervention would ameliorate the 27-OHC-induced colonic occludin (*p* < 0.0001) and claudin-1 (*p* < 0.0001) protein expression level downregulation (Figure 12B,C).

**Figure 13:**
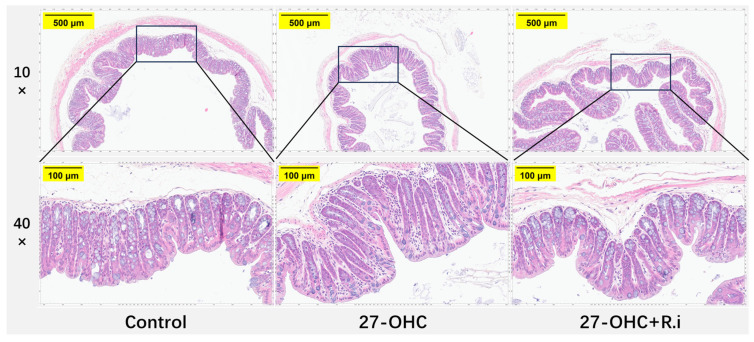
HE staining of colon tissues in C57BL/6J mice in each group. 10× (scale bar = 500 μm), 40× (scale bar = 100 μm).

The results of HE staining of colon tissue showed that, in the control group, the columnar epithelium was neatly arranged, the intramucosal crypts were similar in size and shape, parallel to each other, and the base of the crypts reached the muscularis mucosa below, while the crypts and the cup cells inside the columnar epithelium were filled with mucus, the lymphocyte counts were low in the mucosal lamina propria, and no inflammatory manifestations were noted. In contrast, the colon of the 27-OHC group had a disorganized arrangement of columnar epithelial cells, and reduced mucus content in goblet cells, the size and shape of the crypts within the mucosa are varied, while the bottom of the crypt did not reach the muscularis mucosa. Besides, a large number of lymphocytes appeared in the lamina propria of the mucosa, with an increase in basal plasma cells and the presence of neutrophils, suggesting inflammation. *Roseburia intestinalis* treatment ameliorated the above manifestations of intestinal barrier damage (Figure 13).

## 4. Discussion

27-OHC, as an independent risk factor for cognitive impairment, a series of studies have been conducted on the mechanism of abnormal levels of 27-OHC leading to AD. The lower abundance of *Roseburia intestinalis* [17], the damaged intestinal barrier function [17], the reduced expression level of synapse-related molecules [44], and the decreased level of m6A methylation caused by 27-OHC intervention have attracted our attention. Among them, *Roseburia intestinalis* gavage could reduce the neuroinflammation of rats [25], intestinal barrier dysfunction is related to the central nervous system [19], synaptic dysfunction is a crucial feature of AD [1], and abnormal m6A modification level engaged in the brain function and cognitive dysfunction. In this study, we investigated whether the reduced m6A methylation level and the impaired learning and memory ability caused by 27-OHC could be reversed by the *Roseburia intestinalis* complement.

This study first observed the changes in the learning and memory ability of mice after intervention. The 27-OHC group showed impaired learning and memory ability compared to the control group, but, after the 27-OHC treated mouse was complemented with *Roseburia intestinalis* for 7 days, t the impairment of learning and memory ability caused by 27-OHC could be reversed by *Roseburia intestinalis*, which means the reduction of *Roseburia intestinalis* could be a key part of 27-OHC’s contribution to cognitive impairment. Through this approach, we investigate the crucial role of *Roseburia intestinalis* in the process of 27-OHC leading to cognitive impairment.

*Roseburia intestinalis* and m6A, are both as important mechanisms of 27-OHC leading to learning and memory ability impairment, whether there is a connection between them remains to be clarified. The study then tested the m6A modification level in the brain cortex of each group, the results indicated that the 27-OHC intervention resulted in the decrease of m6A modification level of total RNA in the brain cortex, and the decrease of m6A modification caused by the 27-OHC treatment could be improved by the *Roseburia intestinalis* complementary. As the results show, there is an association between *Roseburia intestinalis* and m6A modification. Besides, the expression level of some m6A modification-related enzyme proteins or genes in the brain cortex were detected by WB and RT-qPCR, finding that several of them did have significantly differential expression levels, which further proved the conclusion that the abnormal change of m6A modification in the brain cortex caused by 27-OHC can be reversed by *Roseburia intestinalis*.

Notably, although the overall level of m6A methylation modification in brain tissue was decreased in cognitive impairment patients [31] and AD animal models [45] compared with the control group, which consistent with the findings of this study that the intervention-induced learning and memory ability changing is positively associated with the changes in m6A modification in brain cortex, regulating the m6A related enzymes level in different cognitive functional units may have diametrically opposite effects on the occurrence and development of cognitive damage. For example, knocking out the METTL3 gene in macrophages derived from monocytes that can cross the blood-brain barrier enhanced the migration of monocytes and accelerated the clearance of Aβ, thereby alleviating AD symptoms [28]. This suggests that the changes in the overall m6A methylation level in the cerebral cortex cannot fully reflect the abnormal changes in the m6A methylation of the cerebral cortex caused by the intervention, and it is necessary to detect the levels of m6A methylation and m6A-related enzymes in different functional units. The results of this study on the protein and gene expression levels of synapse-related molecules such as PSD-95 and BDNF showed that the expression levels of some synapse-related molecules did change with the change of m6A modification levels, however, it is still unclear how abnormal changes in m6A modification lead to synaptic pathology and neurodegenerative diseases [36]. The mechanism by which m6A modification affects synaptic function requires the continued efforts of a wide range of scholars. In addition, although m6A methylation modifications are the most abundant internal modifications in mRNAs, mRNAs account for less than 5% of total RNAs [46]. The modification is also present in RNAs such as tRNAs, mt-RNAs, rRNAs, and long-stranded noncoding [36], suggesting the complexity of m6A modifications involved in the course of AD. The present study only superficially verified the causal association of the abnormal change of m6A modification level of total RNA and its related enzymes in the brain with the 27-OHC intervention, and the *Roseburia intestinalis* supplementation could reverse the abnormal change of m6A methylation in the brain cortex caused by 27-OHC, but the in-depth mechanism still needs to be further explored. In addition to isolating and purifying mRNA in total RNA to detect the m6A methylation level of mRNA in the cerebral cortex, conducting MeRIP-seq sequencing in population studies to map the m6A location transcriptome-wide under the influence of abnormal levels of 27-OHC is also necessary, then carrying out a series of mechanism validation studies to verify whether the screened genes participate in the mechanism of 27-OHC leading to AD. Besides, the non-coding RNAs, including the lncRNA, miRNA, and circRNA, which are related to AD progression [47,48,49], whether their own m6A modification and their interactions with the m6A modifying genes are a part of the mechanisms 27-OHC leading to AD needs in-depth research.

The mechanism by which treatment with *Roseburia intestinalis* can reverse the learning and memory impairment and the decline of m6A modification levels caused by 27-OHC still needs to be further clarified. This study determined that *Roseburia intestinalis* intervention could improve 27-OHC-induced oxysterol including 27-OHC and 24S-OHC metabolic disorders in the brain and intestinal barrier damage, while intestinal barrier damage is an important risk factor in neurodegenerative diseases [19]. These changes may partially reveal the mechanism by which *Roseburia intestinalis* supplementation reverses 27-OHC-induced impairment of learning and memory function and decreased levels of m6A modification. However, it is not enough to fully explain the problem. *Roseburia intestinalis*, as a promising probiotic, may have beneficial effects on cognitive function through a variety of mechanisms. The main or specific metabolites of *Roseburia* spp., including propionate, butyrate, and indoleamine2,3-dioxygenase-1, etc., had shown colonic and systemic anti-inflammatory effects [26,50,51,52,53,54]. Treating mice with the *Roseburia* spp. flagellin alone could also cause beneficial effects such as mitigating colonic inflammation or relieving alcoholic fatty liver symptoms [53,54,55,56]. While both the metabolite and the special structure of this bacterium show positive impacts such as anti-inflammation, the *Roseburia* spp. supplementation also shows an impact on the level of neurotransmitters in the gut or brain like 5-HT (serotonin) and melatonin [25,57], which participate in the gut-brain axis [58,59]. These findings provide us with new directions for further research on the mechanism of *Roseburia intestinalis* supplementation alleviating 27-OHC-induced impairment of learning and memory ability.

## 5. Conclusions

The present study sheds light on the role of 27-OHC and *Roseburia intestinalis* in the m6A methylation modification level, and the pathogenesis of learning and memory ability, highlighting that 27-OHC causes a disturbance in the expression of m6A methylation-related enzymes and reduces the m6A methylation modification level in the brain cortex, and finally leads to learning and memory impairment in mice; however, the *Roseburia intestinalis* supplementation could reverse the negative effects mentioned above. This research could help reveal the mechanism of 27-OHC-induced neural damage and provide important scientific evidence for the future use of *Roseburia intestinalis* in neuroprotection at the animal study level.

## Figures and Tables

**Table 1:** MRM transitions for oxysterols.

Analyte	Quantifier Transition	Qualifier Transition	DP	CE	Retention Time (min)
27-OHC	307 → 124	307 → 490.2	130	20, 10	9.6
27-OHC-D5	309.6 → 124	309.6 → 495.2	130	20, 10	9.37
24S-OHC	307 → 124	307 → 490	130	20, 10	8.87
24S-OHC-D7	310.7 → 124	310.7 → 497.3	130	20, 10	8.81

**Table 2:** Primers used in this study.

Primer	Forward Sequence (5′–3′)	Reverse Sequence (5′–3′)
METTL4	GAAAGGATGGAGGCCAGGAC	TCTCGACAGCCTCTCCTACC
METTL14	CTGAGAGTG CGGATAGCATTG	GAGCAGATGTATCATAGGAAGCC
FTO	TCACAGACGTGGTTTCCGAG	ACCACTGGGTTGAGAGGAGT
WTAP	TAATGGCGAAGTGTCGAATG	CTGCTGTCGTGTCTCCTTCA
YTHDF1	CTGCAGTTAAGACGGTGGGT	TAGCAATGGCTGCCCATGAA
BDNF	TAACGGCGGCAGACAAAAAGA	GAAGTATTGCTTCAGTTGGCCT
PSD-95	CGATTACCACTTTGTCTCCTCCC	ACGGATGAAGATGGCGATAGG
β-actin	ATGACCCAAGCCGAGAAGG	TGCAATGACGTGAGGAACACT

## Data Availability

The data presented in this study are available on request from the corresponding author due to privacy.

## References

[1] Alzheimer’s Association (2023). 2023 Alzheimer’s disease facts and figures. Alzheimer’s Dement..

[2] Self W.K., Holtzman D.M. (2023). Emerging diagnostics and therapeutics for Alzheimer disease. Nat. Med..

[3] Howard R., Kales H.C. (2023). New treatments for Alzheimer’s disease. BMJ.

[4] Li D., Zhang J., Liu Q. (2022). Brain cell type-specific cholesterol metabolism and implications for learning and memory. Trends Neurosci..

[5] Son Y., Yeo I.-J., Hong J.-T., Eo S.-K., Lee D., Kim K. (2023). Side-Chain Immune Oxysterols Induce Neuroinflammation by Activating Microglia. Int. J. Mol. Sci..

[6] Martins G.L., Ferreira C.N., Palotás A., Rocha N.P., Reis H.J. (2023). Role of Oxysterols in the Activation of the NLRP3 Inflammasome as a Potential Pharmacological Approach in Alzheimer’s Disease. Curr. Neuropharmacol..

[7] Sorboni S.G., Moghaddam H.S., Jafarzadeh-Esfehani R., Soleimanpour S. (2022). A Comprehensive Review on the Role of the Gut Microbiome in Human Neurological Disorders. Clin. Microbiol. Rev..

[8] Bairamian D., Sha S., Rolhion N., Sokol H., Dorothée G., Lemere C.A., Krantic S. (2022). Microbiota in neuroinflammation and synaptic dysfunction: A focus on Alzheimer’s disease. Mol. Neurodegener..

[9] Zhu G., Zhao J., Zhang H., Wang G., Chen W. (2023). Gut Microbiota and its Metabolites: Bridge of Dietary Nutrients and Alzheimer’s Disease. Adv. Nutr..

[10] Migliore L., Coppedè F. (2022). Gene-environment interactions in Alzheimer disease: The emerging role of epigenetics. Nat. Rev. Neurol..

[11] Maity S., Farrell K., Navabpour S., Narayanan S.N., Jarome T.J. (2021). Epigenetic Mechanisms in Memory and Cognitive Decline Associated with Aging and Alzheimer’s Disease. Int. J. Mol. Sci..

[12] Jeremic D., Jiménez-Díaz L., Navarro-López J.D. (2023). Targeting epigenetics: A novel promise for Alzheimer’s disease treatment. Ageing Res. Rev..

[13] Merino-Serrais P., Loera-Valencia R., Rodriguez-Rodriguez P., Parrado-Fernandez C., Ismail M.A., Maioli S., Matute E., Jimenez-Mateos E.M., Björkhem I., DeFelipe J. (2019). 27-Hydroxycholesterol Induces Aberrant Morphology and Synaptic Dysfunction in Hippocampal Neurons. Cereb. Cortex.

[14] Wang L., Yu H., Hao L., Ju M., Feng W., Xiao R. (2023). The Interaction Effect of 27-Hydroxycholesterol Metabolism Disorder and CYP27A1 Single Nucleotide Polymorphisms in Mild Cognitive Impairment: Evidence from a Case-Control Study. Mol. Nutr. Food Res..

[15] Liu Q., An Y., Yu H., Lu Y., Feng L., Wang C., Xiao R. (2016). Relationship between oxysterols and mild cognitive impairment in the elderly: A case-control study. Lipids Health Dis..

[16] Wang T., Cui S., Hao L., Liu W., Wang L., Ju M., Feng W., Xiao R. (2022). Regulation of Th17/Treg Balance by 27-Hydroxycholesterol and 24S-Hydroxycholesterol Correlates with Learning and Memory Ability in Mice. Int. J. Mol. Sci..

[17] Wang Y., An Y., Ma W., Yu H., Lu Y., Zhang X., Wang Y., Liu W., Wang T., Xiao R. (2020). 27-Hydroxycholesterol contributes to cognitive deficits in APP/PS1 transgenic mice through microbiota dysbiosis and intestinal barrier dysfunction. J. NeuroInflamm..

[18] Chandra S., Sisodia S.S., Vassar R.J. (2023). The gut microbiome in Alzheimer’s disease: What we know and what remains to be explored. Mol. Neurodegener..

[19] Pellegrini C., Fornai M., D’Antongiovanni V., Antonioli L., Bernardini N., Derkinderen P. (2023). The intestinal barrier in disorders of the central nervous system. Lancet Gastroenterol. Hepatol..

[20] Choi H., Mook-Jung I. (2023). Functional effects of gut microbiota-derived metabolites in Alzheimer’s disease. Curr. Opin. Neurobiol..

[21] Jin J., Xu Z., Zhang L., Zhang C., Zhao X., Mao Y., Zhang H., Liang X., Wu J., Yang Y. (2023). Gut-derived β-amyloid: Likely a centerpiece of the gut-brain axis contributing to Alzheimer’s pathogenesis. Gut Microbes.

[22] Hao L., Wang L., Ju M., Feng W., Guo Z., Sun X., Xiao R. (2023). 27-Hydroxycholesterol impairs learning and memory ability via decreasing brain glucose uptake mediated by the gut microbiota. Biomed. Pharmacother..

[23] Dai D., Zhu J., Sun C., Li M., Liu J., Wu S., Ning K., He L.-J., Zhao X.-M., Chen W.-H. (2022). GMrepo v2: A curated human gut microbiome database with special focus on disease markers and cross-dataset comparison. Nucleic Acids Res..

[24] Verhaar B.J.H., Hendriksen H.M.A., de Leeuw F.A., Doorduijn A.S., van Leeuwenstijn M., Teunissen C.E., Barkhof F., Scheltens P., Kraaij R., van Duijn C.M. (2021). Gut Microbiota Composition Is Related to AD Pathology. Front. Immunol..

[25] Xu F., Cheng Y., Ruan G., Fan L., Tian Y., Xiao Z., Chen D., Wei Y. (2021). New pathway ameliorating ulcerative colitis: Focus on Roseburia intestinalis and the gut-brain axis. Ther. Adv. Gastroenterol..

[26] Song L., Sun Q., Zheng H., Zhang Y., Wang Y., Liu S., Duan L. (2022). Roseburia hominis Alleviates Neuroinflammation via Short-Chain Fatty Acids through Histone Deacetylase Inhibition. Mol. Nutr. Food Res..

[27] Hao Z., Meng C., Li L., Feng S., Zhu Y., Yang J., Han L., Sun L., Lv W., Figeys D. (2023). Positive mood-related gut microbiota in a long-term closed environment: A multiomics study based on the “Lunar Palace 365” experiment. Microbiome.

[28] Yin H., Ju Z., Zheng M., Zhang X., Zuo W., Wang Y., Ding X., Zhang X., Peng Y., Li J. (2023). Loss of the m6A methyltransferase METTL3 in monocyte-derived macrophages ameliorates Alzheimer’s disease pathology in mice. PLoS Biol..

[29] Xia L., Zhang F., Li Y., Mo Y., Zhang L., Li Q., Luo M., Hou X., Du Z., Deng J. (2023). A new perspective on Alzheimer’s disease: m6A modification. Front. Genet..

[30] Boulias K., Greer E.L. (2023). Biological roles of adenine methylation in RNA. Nat. Rev. Genet..

[31] Castro-Hernández R., Berulava T., Metelova M., Epple R., Peña Centeno T., Richter J., Kaurani L., Pradhan R., Sakib M.S., Burkhardt S. (2023). Conserved reduction of m6A RNA modifications during aging and neurodegeneration is linked to changes in synaptic transcripts. Proc. Natl. Acad. Sci. USA.

[32] Kivisäkk P., Carlyle B.C., Sweeney T., Quinn J.P., Ramirez C.E., Trinbetta B.A., Mendes M., Brock M., Rubel C., Czerkowicz J. (2022). Increased levels of the synaptic proteins PSD-95, SNAP-25, and neurogranin in the cerebrospinal fluid of patients with Alzheimer’s disease. Alzheimer’s Res. Ther..

[33] Wang C., Kavalali E., Monteggia L. (2022). BDNF Signaling in Context: From Synaptic Regulation to Psychiatric Disorders. Cell.

[34] Chang R., Zhu S., Peng J., Lang Z., Zhou X., Liao H., Zou J., Zeng P., Tan S. (2023). The hippocampal FTO-BDNF-TrkB pathway is required for novel object recognition memory reconsolidation in mice. Transl. Psychiatry.

[35] Merkurjev D., Hong W.-T., Iida K., Oomoto I., Goldie B.J., Yamaguti H., Ohara T., Kawaguchi S.-Y., Hirano T., Martin K.C. (2018). Synaptic N6-methyladenosine (m6A) epitranscriptome reveals functional partitioning of localized transcripts. Nat. Neurosci..

[36] .Knight H.M., Öz M.D., PerezGrovas-Saltijeral A. (2024). Dysregulation of RNA modification systems in clinical populations with neurocognitive disorders. Neural Regen. Res..

[37] Wellman A.S., Metukuri M.R., Kazgan N., Xu X., Xu Q., Ren N.S.X., Czopik A., Shanahan M.T., Kang A., Chen W. (2017). Intestinal Epithelial Sirtuin 1 Regulates Intestinal Inflammation during Aging in Mice by Altering the Intestinal Microbiota. Gastroenterology.

[38] Cornuault J.K., Moncaut E., Loux V., Mathieu A., Sokol H., Petit M.-A., De Paepe M. (2020). The enemy from within: A prophage of Roseburia intestinalis systematically turns lytic in the mouse gut, driving bacterial adaptation by CRISPR spacer acquisition. ISME J..

[39] Mehla J., Lacoursiere S., Stuart E., McDonald R.J., Mohajerani M.H. (2018). Gradual Cerebral Hypoperfusion Impairs Fear Conditioning and Object Recognition Learning and Memory in Mice: Potential Roles of Neurodegeneration and Cholinergic Dysfunction. J. Alzheimer’s Dis..

[40] Bak J., Pyeon H.-I., Seok J.-I., Choi Y.-S. (2017). Effect of rotation preference on spontaneous alternation behavior on Y maze and introduction of a new analytical method, entropy of spontaneous alternation. Behav. Brain Res..

[41] Wang T., Feng W., Ju M., Yu H., Guo Z., Sun X., Yang K., Liu M., Xiao R. (2023). 27-hydroxycholesterol causes cognitive deficits by disturbing Th17/Treg balance and the related immune responses in mild cognitive impairment patients and C57BL/6J mice. J. Neuroinflamm..

[42] Sidhu R., Jiang H., Farhat N.Y., Carrillo-Carrasco N., Woolery M., Ottinger E., Porter F.D., Schaffer J.E., Ory D.S., Jiang X. (2015). A validated LC-MS/MS assay for quantification of 24(S)-hydroxycholesterol in plasma and cerebrospinal fluid. J. Lipid Res..

[43] Gamba P., Giannelli S., Staurenghi E., Testa G., Sottero B., Biasi F., Poli G., Leonarduzzi G. (2021). The Controversial Role of 24-S-Hydroxycholesterol in Alzheimer’s Disease. Antioxidants.

[44] Wang Y., An Y., Zhang D., Yu H., Zhang X., Wang Y., Tao L., Xiao R. (2019). 27-Hydroxycholesterol Alters Synaptic Structural and Functional Plasticity in Hippocampal Neuronal Cultures. J. Neuropathol. Exp. Neurol..

[45] Shafik A.M., Zhang F., Guo Z., Dai Q., Pajdzik K., Li Y., Kang Y., Yao B., Wu H., He C. (2021). N6-methyladenosine dynamics in neurodevelopment and aging, and its potential role in Alzheimer’s disease. Genome Biol..

[46] Feng Y.-J., You X.-J., Ding J.-H., Zhang Y.-F., Yuan B.-F., Feng Y.-Q. (2022). Identification of Inosine and 2′-O-Methylinosine Modifications in Yeast Messenger RNA by Liquid Chromatography-Tandem Mass Spectrometry Analysis. Anal. Chem..

[47] Wang X., Xie J., Tan L., Lu Y., Shen N., Li J., Hu H., Li H., Li X., Cheng L. (2023). N6-methyladenosine-modified circRIMS2 mediates synaptic and memory impairments by activating GluN2B ubiquitination in Alzheimer’s disease. Transl. Neurodegener..

[48] Su L., Li R., Zhang Z., Liu J., Du J., Wei H. (2022). Identification of altered exosomal microRNAs and mRNAs in Alzheimer’s disease. Ageing Res. Rev..

[49] Balusu S., Horré K., Thrupp N., Craessaerts K., Snellinx A., Serneels L., T’Syen D., Chrysidou I., Arranz A.M., Sierksma A. (2023). MEG3 activates necroptosis in human neuron xenografts modeling Alzheimer’s disease. Science.

[50] Nie K., Ma K., Luo W., Shen Z., Yang Z., Xiao M., Tong T., Yang Y., Wang X. (2021). Roseburia intestinalis: A Beneficial Gut Organism From the Discoveries in Genus and Species. Front. Cell. Infect. Microbiol..

[51] Kang X., Liu C., Ding Y., Ni Y., Ji F., Lau H.C.H., Jiang L., Sung J.J., Wong S.H., Yu J. (2023). Roseburia intestinalis generated butyrate boosts anti-PD-1 efficacy in colorectal cancer by activating cytotoxic CD8+ T cells. Gut.

[52] Kasahara K., Krautkramer K.A., Org E., Romano K.A., Kerby R.L., Vivas E.I., Mehrabian M., Denu J.M., Bäckhed F., Lusis A.J. (2018). Interactions between Roseburia intestinalis and diet modulate atherogenesis in a murine model. Nat. Microbiol..

[53] Seo B., Jeon K., Moon S., Lee K., Kim W.-K., Jeong H., Cha K.H., Lim M.Y., Kang W., Kweon M.-N. (2020). *Roseburia* spp. Abundance Associates with Alcohol Consumption in Humans and Its Administration Ameliorates Alcoholic Fatty Liver in Mice. Cell Host Microbe.

[54] Ruan G., Chen M., Chen L., Xu F., Xiao Z., Yi A., Tian Y., Ping Y., Lv L., Cheng Y. (2022). Roseburia intestinalis and Its Metabolite Butyrate Inhibit Colitis and Upregulate TLR5 through the SP3 Signaling Pathway. Nutrients.

[55] Wu X., Pan S., Luo W., Shen Z., Meng X., Xiao M., Tan B., Nie K., Tong T., Wang X. (2020). Roseburia intestinalis-derived flagellin ameliorates colitis by targeting miR-223-3p-mediated activation of NLRP3 inflammasome and pyroptosis. Mol. Med. Rep..

[56] Patterson A.M., Mulder I.E., Travis A.J., Lan A., Cerf-Bensussan N., Gaboriau-Routhiau V., Garden K., Logan E., Delday M.I., Coutts A.G.P. (2017). Human Gut Symbiont Roseburia hominis Promotes and Regulates Innate Immunity. Front. Immunol..

[57] Song L., He M., Sun Q., Wang Y., Zhang J., Fang Y., Liu S., Duan L. (2021). Roseburia hominis Increases Intestinal Melatonin Level by Activating p-CREB-AANAT Pathway. Nutrients.

[58] Zhang Z., Peng Q., Huo D., Jiang S., Ma C., Chang H., Chen K., Li C., Pan Y., Zhang J. (2021). Melatonin Regulates the Neurotransmitter Secretion Disorder Induced by Caffeine through the Microbiota-Gut-Brain Axis in Zebrafish (*Danio rerio*). Front. Cell Dev. Biol..

[59] Mittal R., Debs L.H., Patel A.P., Nguyen D., Patel K., O’Connor G., Grati M., Mittal J., Yan D., Eshraghi A.A. (2017). Neurotransmitters: The Critical Modulators Regulating Gut-Brain Axis. J. Cell Physiol..

